# A quantitative structure–activity relationship study of anti-HIV activity of substituted HEPT using nonlinear models

**DOI:** 10.1007/s00044-013-0525-4

**Published:** 2013-02-27

**Authors:** Hadi Noorizadeh, Sami Sajjadifar, Abbas Farmany

**Affiliations:** 1 Department of Chemistry, Faculty of Sciences, Islamic Azad University, Ilam Branch, Ilam, Iran; 2 Department of Chemistry, Payame Noor University, PO BOX 19395-3697, Tehran, Iran

**Keywords:** AIDS, Anti-HIV activity, HEPT ligands, QSAR, Genetic algorithm, Levenberg–Marquardt artificial neural network

## Abstract

We performed studies on extended series of 79 HEPT ligands (1-[(2-hydroxyethoxy)methyl]-6-(phenylthio)thymine), inhibitors of HIV reverse-transcriptase with anti-HIV biological activity, using quantitative structure–activity relationship (QSAR) methods that imply analysis of correlations and representation of models. A suitable set of molecular descriptors was calculated, and the genetic algorithm was employed to select those descriptors which resulted in the best-fit models. The kernel partial least square and Levenberg–Marquardt artificial neural network were utilized to construct the nonlinear QSAR models. The proposed methods will be of great significance in this research, and would be expected to apply to other similar research fields.

## Introduction

Acquired immune deficiency syndrome or acquired immunodeficiency syndrome (AIDS) is a disease of the human immune system caused by the human immunodeficiency virus (HIV). This condition would progressively reduce the effectiveness of the immune system and leaves individuals susceptible to opportunistic infections and tumors (Jabs, 2011; Chitra *et al.*, 2011; Ganguli *et al*., 2012; Holland *et al*., 2010; Wachira and Ruger, 2011).

Acquired immunodeficiency syndrome is now a pandemic, and it has been the sixth leading cause of death among people aged 25–44 in the United States since 1995. The World Health Organization estimated that more than 25 million people worldwide have died from this infection since the start of the epidemic (Kallings, 2008). In 2009, AVERT reported that there were 33.3 million people worldwide living with HIV/AIDS, with 2.6 million new HIV infections per year and 1.8 million annual deaths due to AIDS. In 2007, UNAIDS estimated that 33.2 million people worldwide had AIDS that year, AIDS killed 2.1 million people in the course of that year, including 330,000 children, and moreover 76 % of those deaths occurred in sub-Saharan Africa. According to UNAIDS 2009 report, we have had 60 million infected people, 25 million deaths, and 14 million orphaned children in southern Africa since the epidemic began (Nagata *et al*., 2011; Furin *et al.*, 2012).

Human immunodeficiency virus (HIV) causes AIDS. The virus attacks the immune system and leaves the body vulnerable to a variety of life-threatening infections and cancers. Common bacteria, yeast, parasites, and viruses which do not ordinarily cause serious diseases in people with healthy immune systems can cause fatal illnesses in people with AIDS.

HIV has been found in saliva, tears, nervous system tissue and spinal fluid, blood, semen (including pre-seminal fluid, which is the liquid that comes out before ejaculation), vaginal fluid, and breast milk. However, only blood, semen, vaginal secretions, and breast milk generally transmits infection to others (Schmidt, 2011). The virus can be spread (transmitted) by sexual contact (including oral, vaginal, and anal sex), blood [via blood transfusions (now extremely rare in the U.S.) or needle sharing], exchange between mother and baby during pregnancy, childbirth, breastfeeding, or other exposures to one of the above bodily fluids; other methods of spreading the virus are rare and include accidental needle injury, artificial insemination with infected donated semen, and organ transplantation with infected organs. AIDS is not transmitted to a person who donates blood or organs. However, HIV can be transmitted to a person receiving blood or organs from an infected donor. To reduce this risk, blood banks and organ donor programs screen donors, blood, and tissues thoroughly (Johnston *et al.*, 2010; Firląg-Burkacka *et al.*, 2009).

Although treatments for AIDS and HIV can slow the course of the disease, there is no known cure or vaccine. Antiretroviral treatment reduces both the mortality and the morbidity of HIV infection, but these drugs are expensive, and routine access to antiretroviral medication is not available in all countries (Guo and Li, 2011; Fomsgaard *et al.*, 2011). Due to the difficulty in treating HIV infection, preventing infection is a key aim in controlling the AIDS pandemic, with health organizations promoting safe sex and needle-exchange programs in attempts to slow the spread of the virus. HIV is transmitted through direct contact of a mucous membrane or the bloodstream with a bodily fluid containing HIV, such as blood, semen, vaginal fluid, preseminal fluid, and breast milk (Self, 2010).

Acquired immunodeficiency syndrome begins with HIV infection. People infected with HIV may have no symptoms for 10 years or longer, but they can still transmit the infection to others during this symptom-free period. If the infection is not detected and treated, the immune system gradually weakens and AIDS develops. People with AIDS also have an increased risk of developing various cancers such as Kaposi’s sarcoma, cervical cancer, and cancers of the immune system known as lymphomas. In addition, people with AIDS often have systemic symptoms of infection like fevers, sweats (particularly at night), swollen glands, chills, weakness, and weight loss (Holmes *et al.*, 2003). The specific opportunistic infections that AIDS patients develop depend, in part, on the prevalence of these infections in the geographic area in which the patient lives. The initial infection with HIV may produce no symptoms: some people, however, do experience flu-like symptoms with fever, rash, sore throat, and swollen lymph nodes, usually 2–4 weeks after contracting the virus. Some people with HIV infection stay symptom-free for years between the time they are exposed to the virus and when they develop AIDS (Lyons *et al.*, 2011).

An anti-HIV agent can exert its biological activity in different stages of the viral life cycle inhibiting them. Studies were limited to those stages and phenomenon that appear during viral replication: viral binding to the target cell, viral fusion with the host cell by viral penetration into the host cell’s membrane, viral uncovering in the host cell, reverse genomic RNA transcription, integration of the new viral DNA into the host cell’s chromosomes, provirus activation producing mRNA, viral detachment from the host cell, and viral maturation.

**Fig. 1:**
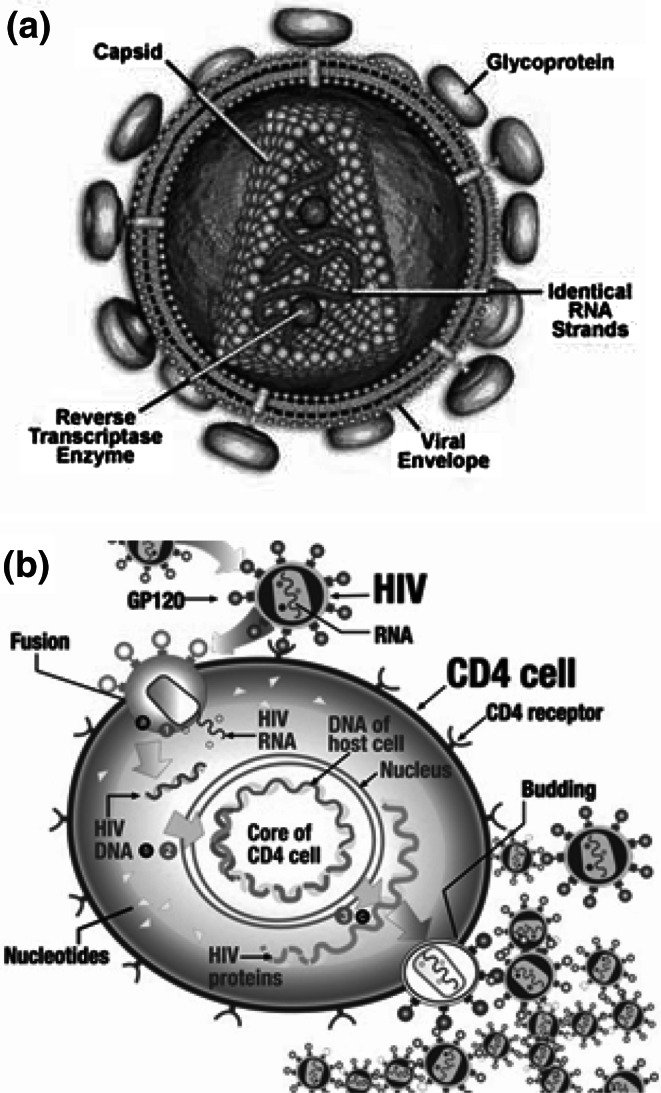
**a** The human immunodeficiency virus (HIV) Anatomy **b** Life cycle of HIV

Reverse transcription of viral genomic RNA into double strained DNA by the RT enzyme is essential for HIV replication. Thus, the inhibition of this essential phase of HIV life cycle provides the most attractive target in order to develop a compound with biological anti-HIV potential. For example, most drugs approved by the FDA for HIV infection treatment are RT inhibitors. High resolution electronic microscopy shows that HIV-1 is a 100 nm virus with a capsule. The external layer is a double lipidic layer derived from the host cell during maturation and contains two major viral glycoproteins (gp): the transmembranar gp41 and outside gp120. There is a protein associated to the membrane (p 18) which provides the matrix for the viral structure and is essential for the integrity of the virus. The matrix surrounds a dense cylindrical characteristic nucleoid which contains the p24 protein from the capside. Inside the nucleoid, there are two identical RNA strains; the viral RNA dependent DNA-polymerase (p66/p55) called reverse-transcriptase (RT) is related to p9 nucleoprotein, to p12 integrase protein, and to components of p15 protease, see Fig. 1 (Ganguli *et al.*, 2012; Wachira and Ruger, 2011; Holmes *et al.*, 2003; Lyon *et al.,* 2011).

**Fig. 2:**
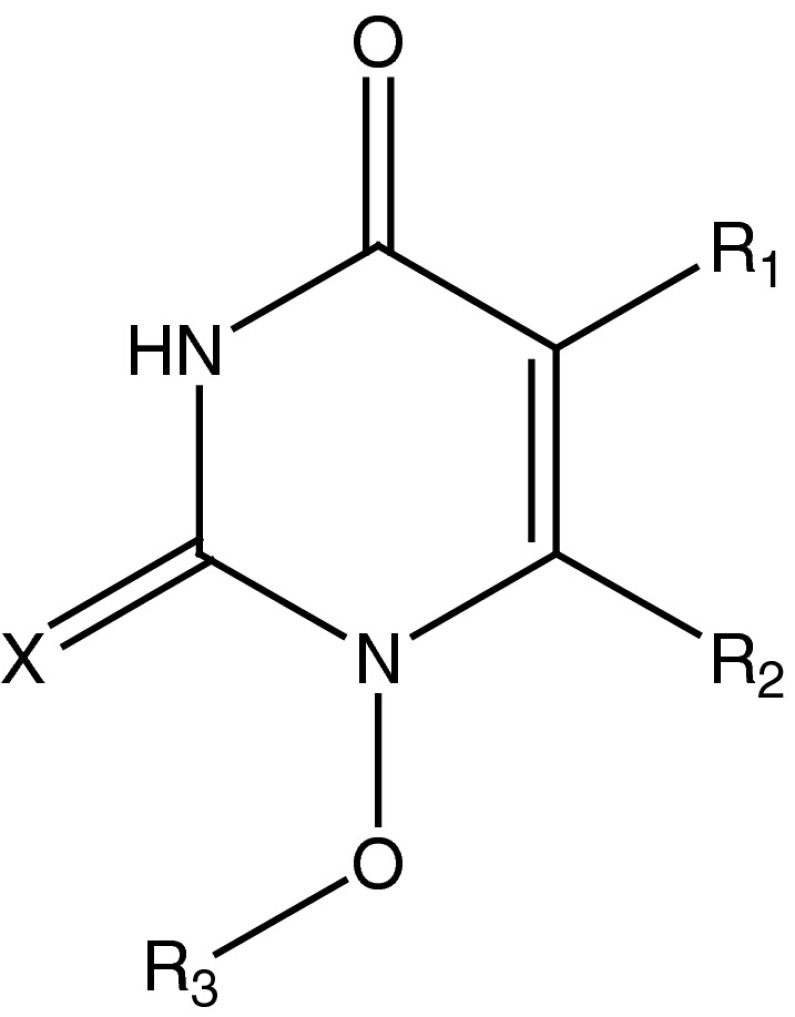
The reference structure of HEPT derivatives

**Fig. 3:**
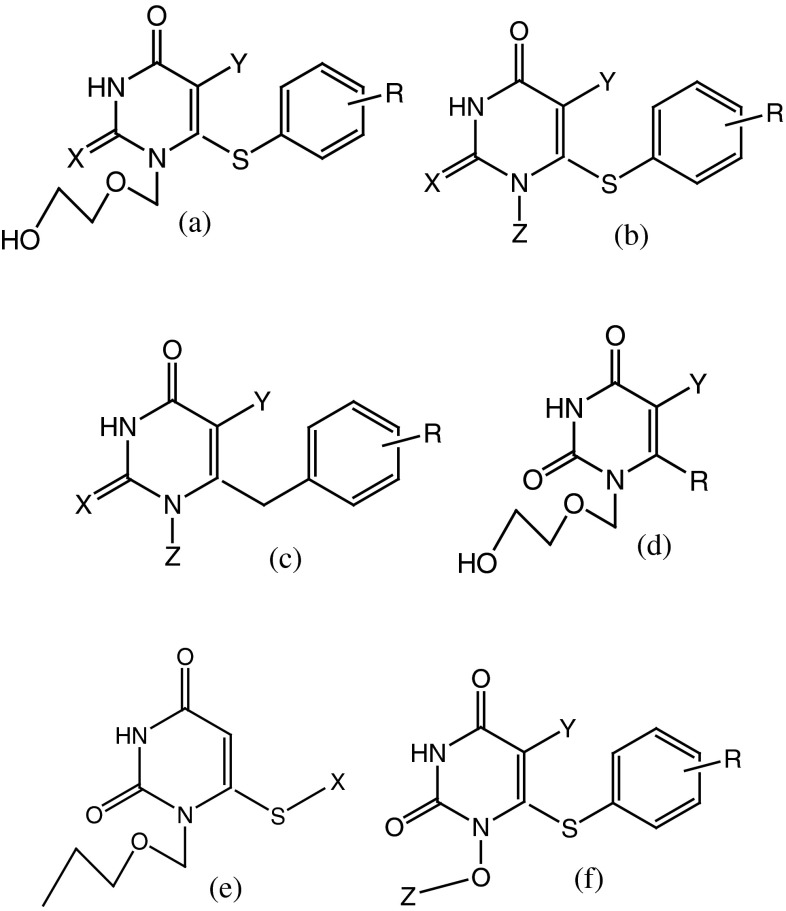
Typical examples of HEPT (1-[(2-hydroxyethoxy)methyl]-6-(phenylthio)thymine) derivatives

By these means, HEPT (1-[(2-hydroxyethoxy)methyl]-6-(phenylthio)thymine) derivatives can be regarded as non-nucleosidic reverse transcriptase inhibitors (NNRTI), see Figs. 2 and 3, and are analogs of the natural substrate. HEPT derivatives don’t interact with the binding site of the DNA or RNA-dependent DNA polymerase. Because of this it is expected that these ligands would not determine side effects. HEPT ligands interact uncompetitively with an allosteric site of the enzyme and don’t affect the substrate binding in a direct way. Actually, NNRTI have a higher binding affinity to the ligand–enzyme complex than to the free enzyme. The HEPT ligand–enzyme interaction leads to enzymatic conformational variations; in other words, the enzyme’s active site has a decreased affinity to the natural substrate. This property is valid only regarding the HIV-1 RT; HEPT ligands are inactive against HIV-2 or other retroviruses. The NNRTI exclusive specificity for the HIV-1 RT is attributed to the presence—at the level of this enzyme (and not in the case of other RT or DNA polymerases)—of a flexible extreme hydrophobic pocket in which HEPT derivatives (different from natural substrate analogs) fit and can be bound (Ji *et al.*, 2007; Wang *et al.*, 2009; Bajaj *et al.*, 2005).

The term *‘half maximal effective concentration*’ (EC_50_) refers to the concentration of a drug, antibody, or toxicant, which induces a response between the baseline and maximum after some specified exposure time. It is commonly used as a measure of a drug’s potency. The EC_50_ of a graded dose–response curve represents the concentration of a compound where 50 % of its maximal effect is observed. The EC_50_ of a quantal dose–response curve represents the concentration of a compound where 50 % of the population exhibits a response, after specified exposure duration (Luis *et al.*, 2010).

Various partial drugs which have been created would treat the HIV infection at various stages but no drug has been found yet to cure. Because of this, we need to comprehend the chemicals and mathematical models that could be applied as an extrapolation model to study the desired features of an anti-HIV drug. The best mathematical model that can quantitatively relate the anti-HIV activity with the structural descriptors is the QSAR model (Quantitative Structure Activity Relationship). The QSAR analysis has been done for various groups of compounds and also for diverse sets of anti-HIV compounds (Goodarzi and Freitas, 2010; Bharate and Singh, 2011; Goodarzi *et al.*, 2009; Si *et al.*, 2008).

There is a trend to develop QSAR from a variety of methods. In particular, genetic algorithm (GA) is frequently used as search algorithm for variable selections in chemometrics and QSAR (Yanmaz *et al.*, 2011). Moreover, nonlinear statistical treatment of QSAR data is expected to provide models with better predictive quality as compared with linear models. In this perspective, artificial neural network (ANN) modeling has become quite common in the QSAR field (Afantitis *et al.*, 2011; Zuperl *et al.*, 2011). Extensive use of ANN, which does not require the “a priori” knowledge of the mathematical form of the relationship between the variables, largely rests on its flexibility (functions of any complexity can be approximated). In recent years, nonlinear kernel-based algorithm as kernel partial least squares (KPLS) has been proposed (Postma *et al.*, 2011). KPLS can efficiently compute latent variables in the feature space by means of nonlinear kernel functions. Compared to other nonlinear PLS methods, the main advantage of the kernel-based algorithm is that it does not involve nonlinear optimization; thus it essentially requires only linear algebra which makes it as simple as the conventional linear PLS. In addition, because of its ability to use different kernel functions, KPLS can handle a wide range of nonlinearities (Cao *et al.*, 2011). In the present study, GA-KPLS and L–M ANN were employed to generate QSAR models that correlate the structure of HEPT ligands and inhibitors of HIV reverse-transcriptase (RT), with anti-HIV biological activity log (1/EC_50_).

## Computational

### Data set

The anti-HIV biological activity log (1/EC_50_) of 79 HEPT derivatives which were taken from the literature (Duda-Seiman *et al.*, 2004) has been presented in Table 1. In this table are given the group of substituents considered on the general structure of Figs. 2 and 3. Biological activities are given as log (1/EC_50_) where EC_50_ represents the concentration and also produces a 50 % protection of MT-4 cells against the direct toxic HIV-1 effect.

**Table 1:** The data set, structure, and the corresponding observed log (1/EC_50_) values

No.	*R*_1_	*R*_2_	*R*_3_	*X*	log (1/EC50)_EXP_
*Calibration set*					
1	Methyl	4-Methylphenylthio	2-Hydroxyethyl	O	3.66
2	Methyl	3-Hydroxyphenylthio	2-Hydroxyethyl	O	4.09
3	Methyl	2-Methylphenylthio	2-Hydroxyethyl	O	4.15
4	Benzyl	Phenylthio	2-Hydroxyethyl	O	4.37
5	Methyl	3-Methoxyphenylthio	2-Hydroxyethyl	O	4.66
6	Methyl	2-Methoxyphenylthio	2-Hydroxyethyl	O	4.72
7	Methyl	3-Tertbutylphenylthio	2-Hydroxyethyl	O	4.92
8	Methyl	3-Cyanophenylthio	2-Hydroxyethyl	O	5.00
9	Methyl	Phenylthio	2-Methoxyethyl	O	5.06
10	Methyl	3-Methoxycarbonylphenylthio	2-Hydroxyethyl	O	5.10
11	Methyl	Phenylthio	2-Benzoyloxyethyl	O	5.12
12	Methyl	Phenylthio	2-Acetyloxyethyl	O	5.17
13	2-Phenylethenyl	Phenylthio	2-Hydroxyethyl	O	5.22
14	Methyl	Phenylthio	2-Azidoethyl	O	5.24
15	Methyl	Phenylthio	Butyl	O	5.33
16	Ethyl	Phenylthio	Cyclohexyl	O	5.40
17	Propyl	Phenylthio	2-Hydroxyethyl	O	5.47
18	Methyl	Phenylthio	Propyl	O	5.48
19	Methyl	3-Ethylphenylthio	2-Hydroxyethyl	O	5.57
20	Allyl	Phenylthio	2-Hydroxyethyl	O	5.60
21	Methyl	Phenylthio	Methyl	O	5.68
22	Ethyl	Phenylthio	Cyclohexyl	S	5.79
23	Methyl	Phenylthio	2-Chloroethyl	O	5.82
24	Methyl	Phenylthio	Propyl	S	5.92
25	Methyl	Phenylthio	2-Hydroxyethyl	S	6.01
26	Ethyl	Phenylthio	Cyclohexylmethyl	O	6.35
27	Ethyl	Phenylthio	Isopropyl	O	6.47
28	Methyl	Phenylthio	Ethyl	O	6.48
29	Methyl	3,5-Dimethylphenylthio	2-Hydroxyethyl	O	6.59
30	Ethyl	Phenylthio	Isopropyl	S	6.66
31	Ethyl	Phenylthio	2-hydroxyethyl	O	6.92
32	Cyclopropyl	Phenylthio	Ethyl	O	7.00
33	Ethyl	Phenylthio	2-Cyclohexylethyl	O	7.02
34	Methyl	Phenylthio	Benzyl	O	7.06
35	Ethyl	Phenylthio	4-Methylbenzyl	S	7.11
36	Isopropyl	Phenylthio	2-Hydroxyethyl	O	7.20
37	Ethyl	3,5-Dichlorophenylthio	2-Hydroxyethyl	S	7.37
38	Ethyl	Phenylthio	Ethyl	S	7.58
39	Ethyl	3,5-Dichlorophenylthio	2-Hydroxyethyl	O	7.85
40	Isopropyl	Phenylthio	Ethyl	S	7.89
41	Ethyl	Phenylthio	4-Chlorobenzyl	S	7.92
42	Ethyl	Phenylthio	Benzyl	S	8.09
43	Ethyl	3,5-Dichlorophenylthio	Ethyl	O	8.13
44	Isopropyl	Phenylthio	Benzyl	S	8.14
45	Ethyl	Phenylthio	Benzyl	O	8.23
46	Isopropyl	3,5-Dimethylphenylthio	2-Hydroxyethyl	S	8.30
47	Isopropyl	Phenylthio	Benzyl	O	8.51
48	Isopropyl	3,5-Dimethylphenylthio	2-Hydroxyethyl	O	8.57
*Prediction set*					
49	Methyl	3-Trifluoromethylphenylthio	2-Hydroxyethyl	O	4.35
50	Methyl	3-Chlorophenylthio	2-Hydroxyethyl	O	4.89
51	Propyl	Phenylthio	2-Hydroxyethyl	S	5.00
52	Methyl	Phenylthio	2-Hydroxyethyl	O	5.15
53	Methyl	3-Fluorophenylthio	2-Hydroxyethyl	O	5.48
54	Methyl	Phenylthio	Methyl	S	5.66
55	Methyl	3,5-Dichlorophenylthio	2-Hydroxyethyl	O	5.89
56	Ethyl	Phenylthio	Cyclohexylmethyl	S	6.45
57	Ethyl	Phenylthio	2-Hydroxyethyl	S	6.96
58	Cyclopropyl	Phenylthio	Ethyl	S	7.02
59	Ethyl	Phenylthio	Ethyl	O	7.72
60	Ethyl	3,5-Dichlorophenylthio	Ethyl	S	7.89
61	Isopropyl	Phenylthio	Ethyl	O	7.99
62	Ethyl	3,5-Dimethylphenylthio	2-Hydroxyethyl	S	8.11
63	Ethyl	3,5-Dimethylphenylthio	Ethyl	O	8.24
64	Ethyl	3,5-Dimethylphenylthio	Benzyl	O	8.55
*Test set*					
65	Methyl	2-Nitrophenylthio	2-Hydroxyethyl	O	3.85
66	Methyl	3-Nitrophenylthio	2-Hydroxyethyl	O	4.47
67	Methyl	3-Iodophenylthio	2-Hydroxyethyl	O	5.00
68	Methyl	3-Acetylphenylthio	2-Hydroxyethyl	O	5.14
69	Methyl	3-Bromophenylthio	2-Hydroxyethyl	O	5.24
70	Iodo	Phenylthio	2-Hydroxyethyl	O	5.44
71	Methyl	3-Methylphenylthio	2-Hydroxyethyl	O	5.59
72	Ethenyl	Phenylthio	2-Hydroxyethyl	O	5.69
73	Methyl	Phenylthio	2-Fluoroethyl	O	5.96
74	Methyl	3,5-Dimethylphenylthio	2-Hydroxyethyl	S	6.66
75	Ethyl	Phenylthio	2-Phenylethyl	S	7.04
76	Isopropyl	Phenylthio	2-Hydroxyethyl	S	7.23
77	Ethyl	3,5-Dimethylphenylthio	2-Hydroxyethyl	O	7.89
78	Ethyl	3,5-Dimethylphenylthio	Benzyl	S	8.14
79	Ethyl	3,5-Dimethylphenylthio	Ethyl	S	8.30

### Computer hardware and software

All calculations were run on a HP laptop computer with an AMD Turion64X2 processor and a Windows XP operating system. The optimizations of molecular structures were done by HyperChem 7.0 and descriptors were calculated by Dragon Version 3.0 software. Cross validation, GA-KPLS, L–M ANN and other calculations were performed in the MATLAB (Version 7, Mathworks, Inc.) environment.

### Molecular modeling and theoretical molecular descriptors

The derivation of theoretical molecular descriptors proceeds from the chemical structure of the compounds. In order to calculate the theoretical descriptors, molecular structures were constructed with the aid of HyperChem version 7.0. The final geometries were obtained with the semi-empirical AM1 method in HyperChem program. The molecular structures were optimized using Fletcher–Reeves algorithm until the root mean square gradient was 0.01 kcal mol^−1^. The resulting geometry was transferred into Dragon program in order to calculate 1,497 descriptors, which was developed by Todeschini *et al.*, (2003).

### Genetic algorithm for descriptor selection

To select the most relevant descriptors with GA, the evolution of the population was simulated (Noorizadeh and Noorizadeh, 2012; Van Dijck and Van Hulle, 2011; Cséfalvayová *et al.*, 2010). Each individual of the population, defined by a chromosome of binary values, represented a subset of descriptors. The number of the genes at each chromosome was equal to the number of the descriptors. The population of the first generation was selected randomly. A gene was given the value of one if its corresponding descriptor was included in the subset; otherwise, it was given the value of zero. The number of the genes with the value of one was kept relatively low to have a small subset of descriptors (Hao *et al.*, 2011); in other words, the probability of generating zero for a gene was set greater. The operators used here were crossover and mutation. The application probability of these operators was varied linearly with a generation renewal. For a typical run, the evolution of the generation was stopped, when 90 % of the generations had taken the same fitness. In this paper, size of the population is 30 chromosomes, the probability of initial variable selection is 5:*V* (*V* is the number of independent variables), crossover is multi Point, the probability of crossover is 0.5, mutation is multi Point, the probability of mutation is 0.01, and the number of evolution generations is 1,000. For each set of data, 3,000 runs were performed.

### Nonlinear model

#### Artificial neural network

**Fig. 4:**
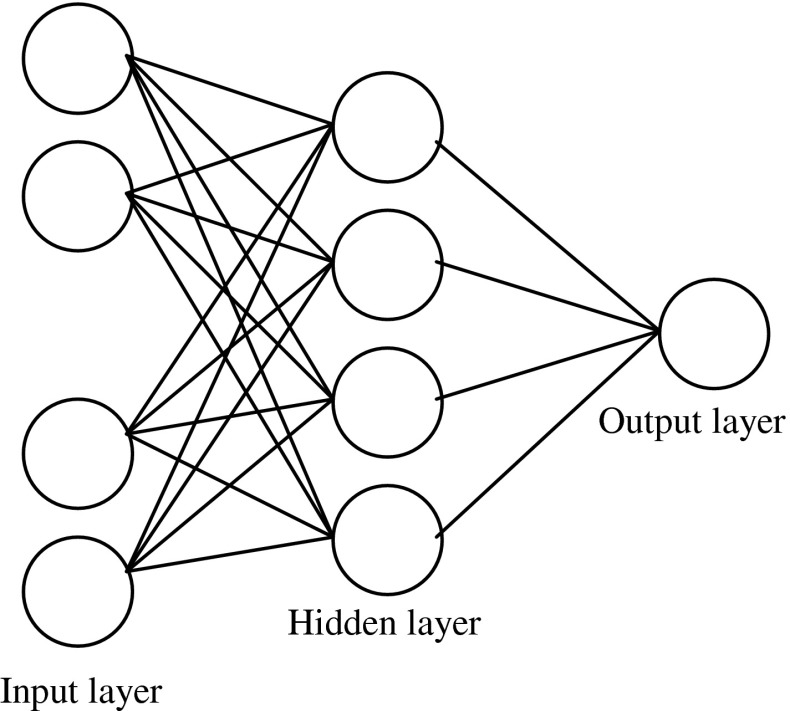
Used three layer ANN

An artificial neural network (ANN) with a layered structure is a mathematical system that stimulates biological neural network consisting of computing units named neurons and connections between neurons named synapses (Noorizadeh and Farmany, 2012; Garkani-Nejad and Ahmadi-Roudi, 2010; Singh *et al.*, 2010). All feed-forward ANN used in this paper are three-layer networks. Each neuron in any layer is fully connected with the neurons of a succeeding layer. Figure 4 shows an example of the architecture of such ANN. The Levenberg–Marquardt back propagation algorithm was used for ANN training and the linear functions were used as the transformation functions in hidden and output layers.

## Results and discussion

### Nonlinear models

#### Results of the GA-KPLS model

**Fig. 5:**
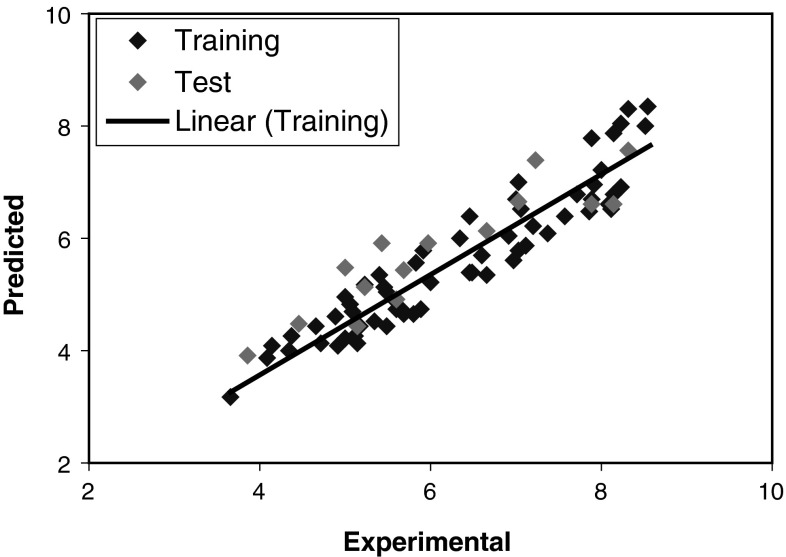
*Plots* of predicted log (1/EC_50_) against the experimental values by GA-KPLS model

The leave-group-out cross validation (LGO-CV) has been performed. In this research, a radial basis kernel function, $k(x,y) = \exp \left( {{{\left| {\left| {x - y} \right|} \right|^{2} } \mathord{\left/ {\vphantom {{\left| {\left| {x - y} \right|} \right|^{2} } c}} \right. \kern-0pt} c}} \right)$, was selected as the kernel function with $c = rm\sigma^{2}$ where *r* is constant that can be determined by considering the process to be predicted (here *r* set to be 1), m is the dimension of the input space, and $\sigma^{2}$ is the variance of the data (Kim *et al.*, 2005). It means that the value of c depends on the system under the study. The 14 descriptors in five latent variables—space chosen by GA-KPLS feature selection methods—were contained constitutional descriptors (number of Oxygen atoms (*n*O) and number of non-H bonds (*n*BO)), topological descriptors (centralization (CENT)), geometric descriptors (gravitational index G2 (bond-restricted) (G2), 3D Petitjean shape index (PJI3), and Qxx COMMA2 value/weighted by atomic van der Waals volumes (QXXv)), 3D-MoRSE descriptors (3D-MoRSE—signal 09/weighted by atomic masses (Mor09m)), WHIM descriptors (first component accessibility directional WHIM index/weighted by atomic polarizabilities (E1p) and A total size index/weighted by atomic electrotopological states (As)), atom-centered fragments (number of terminal primary C(sp^3^) (*n*Cp), CH3R/CH4 (C-001) and phenol/enol/carboxyl OH (O-057)) and charge descriptors (relative positive charge (RPCG) and submolecular polarity parameter (SPP)). The *R* ^2^ and RE for training and test sets were (0.861, 0.748) and (14.37, 23.09), respectively. For the constructed model, two general statistical parameters were selected to evaluate the prediction ability of the model for the log (1/EC_50_). The predicted values of log (1/EC_50_) are plotted against the experimental values for training and test sets in Fig. 5. Consequently, as a result, the number of components (latent variables) is less than the number of independent variables in KPLS analysis. The statistical parameters highest square correlation coefficient leave-group-out cross validation (*R* ^2^) and relative error (RE) were obtained for proposed models. Each of the statistical parameters mentioned above was used for assessing the statistical significance of the QSAR model. This GA-KPLS approach currently constitutes the most accurate method for predicting the anti-HIV biological activity of the drug compounds. The KPLS model uses higher number of descriptors that allows the model to extract better structural information from descriptors to result in a lower prediction error. This suggests that GA-KPLS holds promise for applications in choosing variables for L–M ANN systems. This result indicates that the log (1/EC_50_) of these drugs possesses some nonlinear characteristics.

#### Results of the L–M ANN model

**Fig. 6:**
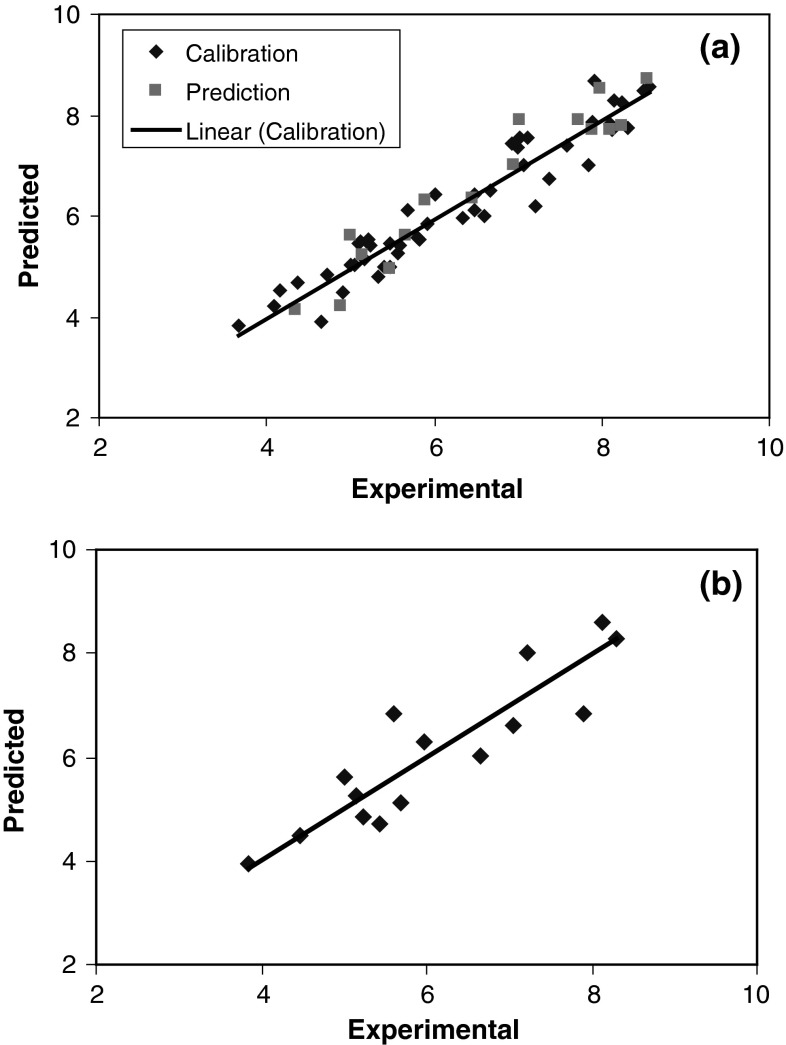
*Plot* of predicted log (1/EC_50_) obtained by L–M ANN against the experimental values **a** calibration and prediction set of molecules and **b** for test set

**Fig. 7:**
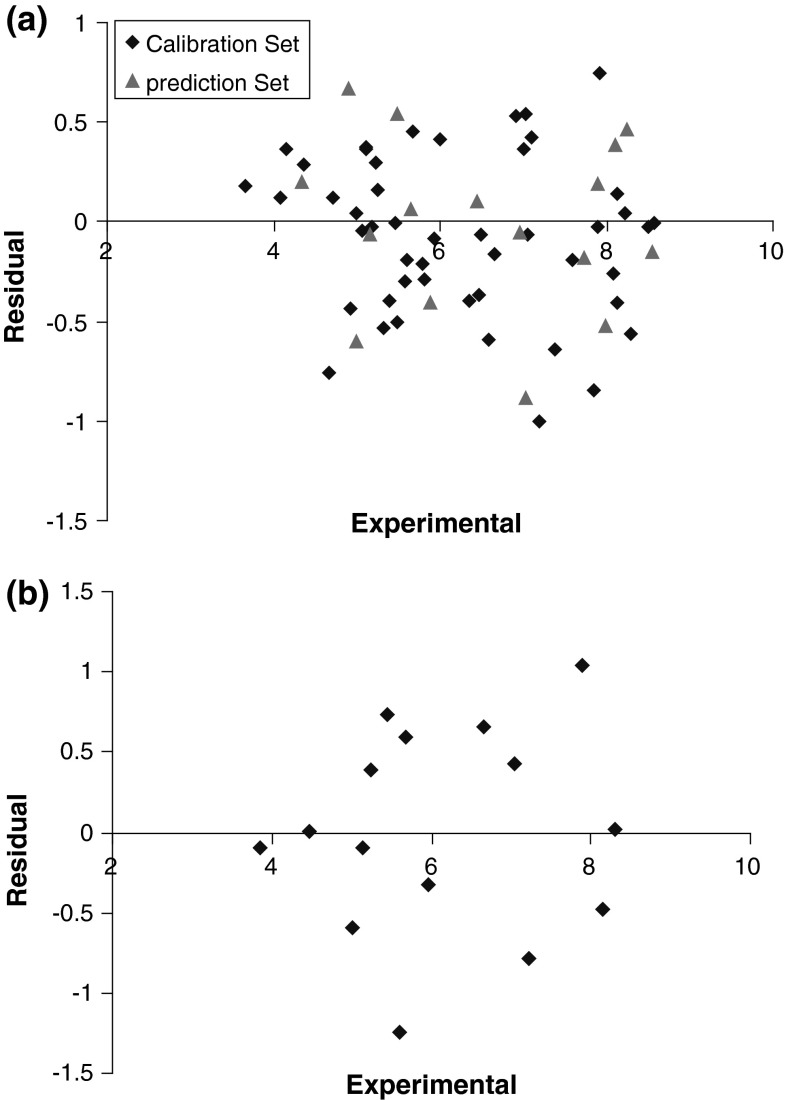
*Plot* of residuals obtained by L–M ANN against the experimental log (1/EC_50_) values **a** training set of molecules and** b** for test set

With the aim of improving the predictive performance of nonlinear QSAR model, L–M ANN modeling was performed. The networks were generated using the 14 descriptors appearing in the GA-KPLS models as their inputs and log (1/EC_50_) as their output. For ANN generation, data set was separated into three groups: calibration, prediction, and test sets. A three-layer network with a sigmoid transfer function was designed for each ANN. Before training the networks, the input and output values were normalized between −1 and 1. Then, the network was trained using the training set and the back propagation strategy for optimizing the weights and bias values. The proper number of nodes in the hidden layer was determined by training the network with different number of nodes in the hidden layer. The root-mean-square error (RMSE) value measures how good the outputs are in comparison with the target values. It should be noted that for evaluating the over fitting, the training of the network for the prediction of log (1/EC_50_) must stop when the RMSE of the prediction set begins to increase while RMSE of calibration set continues to decrease. Therefore, training the network was stopped when overtraining began. All of the above mentioned steps were carried out using basic back propagation, conjugate gradient, and Levenberge–Marquardt weight update functions. Accordingly, one can realize that the RMSE for the training and test sets are minimum when five neurons were selected in the hidden layer. Finally, the number of iterations was optimized with the optimum values for the variables. The R^2^ and RE for calibration, prediction, and test sets were (0.916, 0.894, 0.868) and (9.98, 11.34, 15.29), respectively. The experimental, calculated, relative error and RMSE values log (1/EC_50_) by L–M ANN are shown in Table 2. Inspection of the results reveals a higher *R* ^2^ and lowers other values parameter for the training, test, and prediction sets compared with their counterparts for GA-KPLS. Plots of predicted log (1/EC_50_) versus experimental log (1/EC_50_) values by L–M ANN for calibration, prediction, and test sets are shown in Fig. 6a, b. Obviously, there is a close agreement between the experimental and predicted log (1/EC_50_), and the data represent a very low scattering around a straight line with respective slope and intercept close to one and zero. This clearly shows the strength of L–M ANN as a nonlinear feature selection method. The key strength of L–M ANN is their ability to allow for flexible mapping of the selected features by manipulating their functional dependence implicitly. The residuals (predicted log (1/EC_50_) − experimental log (1/EC_50_)) obtained by the L–M ANN modeling versus the experimental log (1/EC_50_) values are shown in Fig. 7a, b. As the calculated residuals are distributed on both sides of the zero line, one may conclude that there is no systematic error in the development of the neural network. The whole of these data clearly displays a significant improvement of the QSAR model consequent to nonlinear statistical treatment.

**Table 2:** Experimental, calculated, relative error, and RMSE values log (1/EC_50_) by L–M ANN model

No.	log (1/EC_50_)_EXP_	log (1/EC_50_)_CAl_	Relative error	Residuals	RMSE
*Calibration set*					
1	3.66	3.84	4.86	0.18	0.03
2	4.09	4.21	3.02	0.12	0.02
3	4.15	4.52	8.80	0.36	0.05
4	4.37	4.66	6.66	0.29	0.04
5	4.66	3.90	16.31	−0.76	0.11
6	4.72	4.84	2.60	0.12	0.02
7	4.92	4.49	8.84	−0.43	0.06
8	5.00	5.04	0.84	0.04	0.01
9	5.06	5.02	0.89	−0.04	0.01
10	5.10	5.47	7.26	0.37	0.05
11	5.12	5.48	7.10	0.36	0.05
12	5.17	5.14	0.56	−0.03	0.00
13	5.22	5.52	5.74	0.30	0.04
14	5.24	5.40	3.12	0.16	0.02
15	5.33	4.80	10.00	−0.53	0.08
16	5.40	5.00	7.38	−0.40	0.06
17	5.47	5.46	0.10	−0.01	0.00
18	5.48	4.97	9.23	−0.51	0.07
19	5.57	5.27	5.45	−0.30	0.04
20	5.60	5.41	3.44	−0.19	0.03
21	5.68	6.13	7.99	0.45	0.07
22	5.79	5.57	3.73	−0.22	0.03
23	5.82	5.53	4.97	−0.29	0.04
24	5.92	5.84	1.34	−0.08	0.01
25	6.01	6.42	6.85	0.41	0.06
26	6.35	5.95	6.31	−0.40	0.06
27	6.47	6.10	5.72	−0.37	0.05
28	6.48	6.42	0.96	−0.06	0.01
29	6.59	6.00	8.95	−0.59	0.09
30	6.66	6.50	2.40	−0.16	0.02
31	6.92	7.45	7.73	0.53	0.08
32	7.00	7.37	5.23	0.37	0.05
33	7.02	7.56	7.68	0.54	0.08
34	7.06	7.00	0.85	−0.06	0.01
35	7.11	7.54	5.98	0.43	0.06
36	7.20	6.20	13.89	−1.00	0.14
37	7.37	6.73	8.69	−0.64	0.09
38	7.58	7.39	2.50	−0.19	0.03
39	7.85	7.00	10.83	−0.85	0.12
40	7.89	7.86	0.32	−0.03	0.00
41	7.92	8.66	9.39	0.74	0.11
42	8.09	7.83	3.16	−0.26	0.04
43	8.13	7.73	4.95	−0.40	0.06
44	8.14	8.28	1.70	0.14	0.02
45	8.23	8.27	0.47	0.04	0.01
46	8.30	7.74	6.73	−0.56	0.08
47	8.51	8.49	0.27	−0.02	0.00
48	8.57	8.56	0.08	−0.01	0.00
*Prediction set*					
49	4.35	4.15	4.58	0.20	0.05
50	4.89	4.22	13.72	0.67	0.17
51	5.00	5.60	12.00	−0.60	0.15
52	5.15	5.21	1.17	−0.06	0.02
53	5.48	4.94	9.94	0.54	0.14
54	5.66	5.60	1.05	0.06	0.01
55	5.89	6.30	6.96	−0.41	0.10
56	6.45	6.34	1.65	0.11	0.03
57	6.96	7.01	0.72	−0.05	0.01
58	7.02	7.90	12.54	−0.88	0.22
59	7.72	7.90	2.33	−0.18	0.05
60	7.89	7.70	2.41	0.19	0.05
61	7.99	8.51	6.51	−0.52	0.13
62	8.11	7.73	4.75	0.39	0.10
63	8.24	7.78	5.56	0.46	0.11
64	8.55	8.70	1.75	−0.15	0.04
*Test set*					
65	3.85	3.95	2.62	−0.10	0.03
66	4.47	4.47	0.11	0.00	0.00
67	5.00	5.60	12.00	−0.60	0.15
68	5.14	5.24	1.95	−0.10	0.03
69	5.24	4.85	7.42	0.39	0.10
70	5.44	4.70	13.61	0.74	0.19
71	5.59	6.84	22.36	−1.25	0.32
72	5.69	5.10	10.37	0.59	0.15
73	5.96	6.29	5.52	−0.33	0.08
74	6.66	6.01	9.79	0.65	0.17
75	7.04	6.62	6.02	0.42	0.11
76	7.23	8.01	10.79	−0.78	0.20
77	7.89	6.85	13.14	1.04	0.27
78	8.14	8.62	5.86	−0.48	0.12
79	8.30	8.28	0.30	0.03	0.01

### Model validation and statistical parameters

The applied internal (leave-group-out cross validation (LGO-CV)) and external (test set) validation methods were used for the predictive power of models. In the leave-group-out procedure, one compound was removed from the data set, the model was trained with the remaining compounds and used to predict the discarded compound. The process was repeated for each compound in the data set. The predictive power of the models developed on the selected training set is estimated on the predicted values of test set chemicals. The data set should be divided into three new sub-data sets, one for calibration and prediction (training), and the other one for testing. The calibration set was used for model generation. The prediction set was applied to deal with overfitting of the network, whereas test set, the molecules of which have no role in model building was used for the evaluation of the predictive ability of the models for external set.

On the other hand by means of training set, the best model is found and then, its prediction power is checked by test set, as an external data set. In this study, from all 79 components, 48 components are in calibration set, 16 components are in prediction set, and 15 components are in test set).

The result clearly displays a significant improvement of the QSAR model consequent to nonlinear statistical treatment and a substantial independence of model prediction from the structure of the test molecule. In the above analysis, the descriptive power of a given model has been measured by its ability to predict partition of unknown drugs.

For the constructed models, some general statistical parameters were selected to evaluate the predictive ability of the models for log (1/EC_50_) values. In this case, the predicted log (1/EC_50_) of each sample in prediction step was compared with the experimental acidity constant. The first statistical parameter was relative error (RE) that shows the predictive ability of each component, and is calculated as:

1 $$ {\text{RE}}\;(\% ) = 100 \times \left[ {\frac{1}{n}\sum\limits_{i = 1}^{n} {\frac{{(y_{i}^{ \wedge } - y_{i} )}}{{y_{i} }}} } \right] $$

The predictive ability was evaluated by the square of the correlation coefficient (*R* ^2^) which is based on the prediction error sum of squares and was calculated by the following equation:

2 $$ R^{2} = \frac{{\sum\limits_{i = 1}^{n} {(y_{i}^{ \wedge } - \bar{y})} }}{{\sum\limits_{i = 1}^{n} {(y_{i} - \bar{y})} }} $$

where *y*_*i*_ is the experimental log (1/EC_50_) in the sample *i*, $y_{i}^{ \wedge }$ represented the predicted log (1/EC_50_) in the sample *i*, $\bar{y}$ is the mean of experimental log (1/EC_50_) in the prediction set and *n* is the total number of samples used in the test set.

The main aim of the present study was to assess the performances of GA-KPLS and L–M ANN for modeling the anti-HIV biological activity of drugs. The procedures of modeling including descriptor generation, splitting of the data, variable selection, and validation were the same as those performed for modeling of the log (1/EC_50_) of HEPT ligands and RT drugs.

## Conclusion

In the current research, two nonlinear methods (GA-KPLS and L–M ANN) were used to construct a quantitative relation between the anti-HIV biological activity of HEPT ligands and RT drugs and their calculated descriptors. The results obtained by L–M ANN were compared with the results obtained by GA-KPLS model. The results demonstrated that L–M ANN was more powerful in the log (1/EC_50_) prediction of the drug compounds than GA-KPLS. A suitable model with high statistical quality and low prediction errors was eventually derived. This model could accurately predict the anti-HIV biological activity of these components that did not exist in the modeling procedure. It was easy to notice that there was a good prospect for the L–M ANN application in the QSAR modeling.
